# Fluorescence Fluctuation Spectroscopy enables quantification of potassium channel subunit dynamics and stoichiometry

**DOI:** 10.1038/s41598-021-90002-2

**Published:** 2021-05-21

**Authors:** Giulia Tedeschi, Lorenzo Scipioni, Maria Papanikolaou, Geoffrey W. Abbott, Michelle A. Digman

**Affiliations:** 1 Department of Biomedical Engineering, Laboratory for Fluorescence Dynamics, University of California Irvine, Irvine, CA 92697 USA; 2 Department of Physiology and Biophysics, Bioelectricity Laboratory, School of Medicine, University of California Irvine, Irvine, CA 92697 USA

**Keywords:** Biophysics, Biotechnology, Nanoscience and technology

## Abstract

Voltage-gated potassium (Kv) channels are a family of membrane proteins that facilitate K^+^ ion diffusion across the plasma membrane, regulating both resting and action potentials. Kv channels comprise four pore-forming α subunits, each with a voltage sensing domain, and they are regulated by interaction with β subunits such as those belonging to the KCNE family. Here we conducted a comprehensive biophysical characterization of stoichiometry and protein diffusion across the plasma membrane of the epithelial KCNQ1-KCNE2 complex, combining total internal reflection fluorescence (TIRF) microscopy and a series of complementary Fluorescence Fluctuation Spectroscopy (FFS) techniques. Using this approach, we found that KCNQ1-KCNE2 has a predominant 4:4 stoichiometry, while non-bound KCNE2 subunits are mostly present as dimers in the plasma membrane. At the same time, we identified unique spatio-temporal diffusion modalities and nano-environment organization for each channel subunit. These findings improve our understanding of KCNQ1-KCNE2 channel function and suggest strategies for elucidating the subunit stoichiometry and forces directing localization and diffusion of ion channel complexes in general.

## Introduction

Potassium ion (K^+^) channels are a numerous and diverse class of membrane proteins that facilitate passage of K^+^ across biological membranes by creating an environment conducive to selective, gated yet rapid passive diffusion down the electrochemical gradient. In higher animals, the K^+^ channels are divided based on their primary structure and functional attributes into the voltage-gated potassium (Kv) channels, the Ca^2+^-activated potassium (K_Ca_) channels, the inward rectifier potassium (K_ir_) channels and the two-pore domain potassium (K2P) channels^1,2^. Kv channels comprise four α subunits each containing a pore module attached to a voltage-sensing domain (VSD); the assembled tetramer contains a single, central pore^2^. Kv channels are opened by depolarization of the cell membrane, a voltage shift sensed by the VSD, that results in a conformational shift which is communicated to the pore which in turn opens^3^. Kv channels are therefore essential for action potential repolarization and thus for the ability to fire trains of action potentials^4^. K2P channels, formed in contrast from dimers of subunits, can remain open at resting voltages and thereby contribute substantially to setting resting membrane potential of many cell types^1^. Kv channels and K2P channels are structurally distinct but exhibit some functional overlap. K2P channels lack the classic VSD but may exhibit non-canonical voltage-gating behavior. By the same token, a small subset of Kv channels can lose or greatly shift their voltage dependence, becoming constitutively active at resting membrane potentials. This property enables them to serve roles in non-excitable cells, such as epithelial cells. The prime example is the KCNQ1 (Kv7.1) α subunit^5^. Homomeric KCNQ1 channels are voltage-gated and closed at resting membrane potential. However, KCNQ1 homomers likely do not play a major role in native K^+^ currents. Instead, KCNQ1 partners with β subunits of the KCNE family to produce a range of currents important in native cellular physiology^6^.

The KCNE subunits are single-pass transmembrane β subunits best known for modifying the functional properties of voltage-gated potassium (Kv) channel α subunits in the auditory epithelium, other epithelia, auditory neurons, and cardiac myocytes^7,8^. Each of the five human KCNE subunits can regulate multiple different Kv channel α subunits, typically forming heteromeric complexes with unique functional attributes compared to those of other subunit compositions. In addition, many of the forty known Kv α subunits in the human genome are regulated by more than one KCNE isoform. This leads to an impressive potential combinatorial complexity^6,9–11^. While KCNQ1-KCNE1 complexes retain their voltage-dependence, albeit their gating is slowed and other attributes changed compared to homomeric KCNQ1, co-assembly of KCNQ1 with KCNE2 or KCNE3 exerts more dramatic influence. KCNE2 regulates KCNQ1 in the gastric, thyroid, choroid plexus and pancreatic epithelia, typically enabling function of various ions and other solute transporters^12–15^. KCNE3 regulates KCNQ1 in the colon epithelium, where the complex provides a basal K^+^ flux to regulate cAMP-stimulated chloride ion secretion^16^, and KCNQ1-KCNE3 complexes are thought to also be expressed in mammary epithelium^17^. All these tasks require persistent (non-inactivating) K^+^ flux at negative membrane potentials, a feature not typically associated with Kv channels. Accordingly, KCNE2 and KCNE3 each convert KCNQ1 to a constitutively active K^+^ channel, facilitating the above essential functions^6^. The past 25 years have yielded a wealth of knowledge surrounding the importance of KCNE-based potassium channels in cells and tissues including auditory epithelia, auditory neurons, other epithelia, and cardiac myocytes.

Further, KCNEs serve broad roles extending beyond direct regulation of Kv channel electrical attributes, including modulating other types of ion channel, and also regulating features of Kv channel biology beyond their electrical attributes—such as trafficking, α subunit composition, and regulation by other proteins and by ions and small molecules including drugs, protons and PIP_2_^9–11,18,19^. Studies of *Kcne* knockout (*Kcne*^−/−^) mice have uncovered crucial and diverse KCNE activities, and predicted disorders that were subsequently linked to human gene disruption^8,12,14,20–25^. In addition, human *KCNE* gene variants are implicated in life-threatening inherited or acquired human disorders. These include cardiac rhythm disturbances such as Long QT syndrome (LQTS), Brugada Syndrome (BrS) and atrial fibrillation (AF)^26^. *Kcne* germline knockout mice exhibit syndromes compatible to those in humans as well as additional ones not yet recognized in human populations. Examples of the former include the inherited arrhythmia syndromes mentioned above and also the recent discovery of an unexpected link between KCNE2 and coronary artery disease in people^27,28^ and in mice^29^. As an example of the latter, *Kcne2* gene deletion in mice causes a multisystem syndrome predisposing to sudden cardiac death that includes multiple risk factors for coronary artery disease, such as diabetes, elevated serum LDL and angiotensin II, fatty liver and also anemia^22,30,31^. Some of the features of this KCNE2-linked multisystem pathology have been recognized in human populations^27,28^. *Kcne2* deletion also causes hypothyroidism, achlorhydria and increased seizure susceptibility in mice^12–14^.

More recently, investigators have made important breakthroughs using high-resolution spectroscopy, cryo-electron microscopy and modeling to elucidate the structure, binding sites and mechanisms of action of KCNEs within Kv channel complexes^32–35^. Despite these studies, some fundamental questions regarding the molecular composition and subunit dynamics of KCNE complexes remain either controversial or incompletely addressed. These questions often lie in the middle ground between high resolution structure and cell biology.

**Figure 1:**
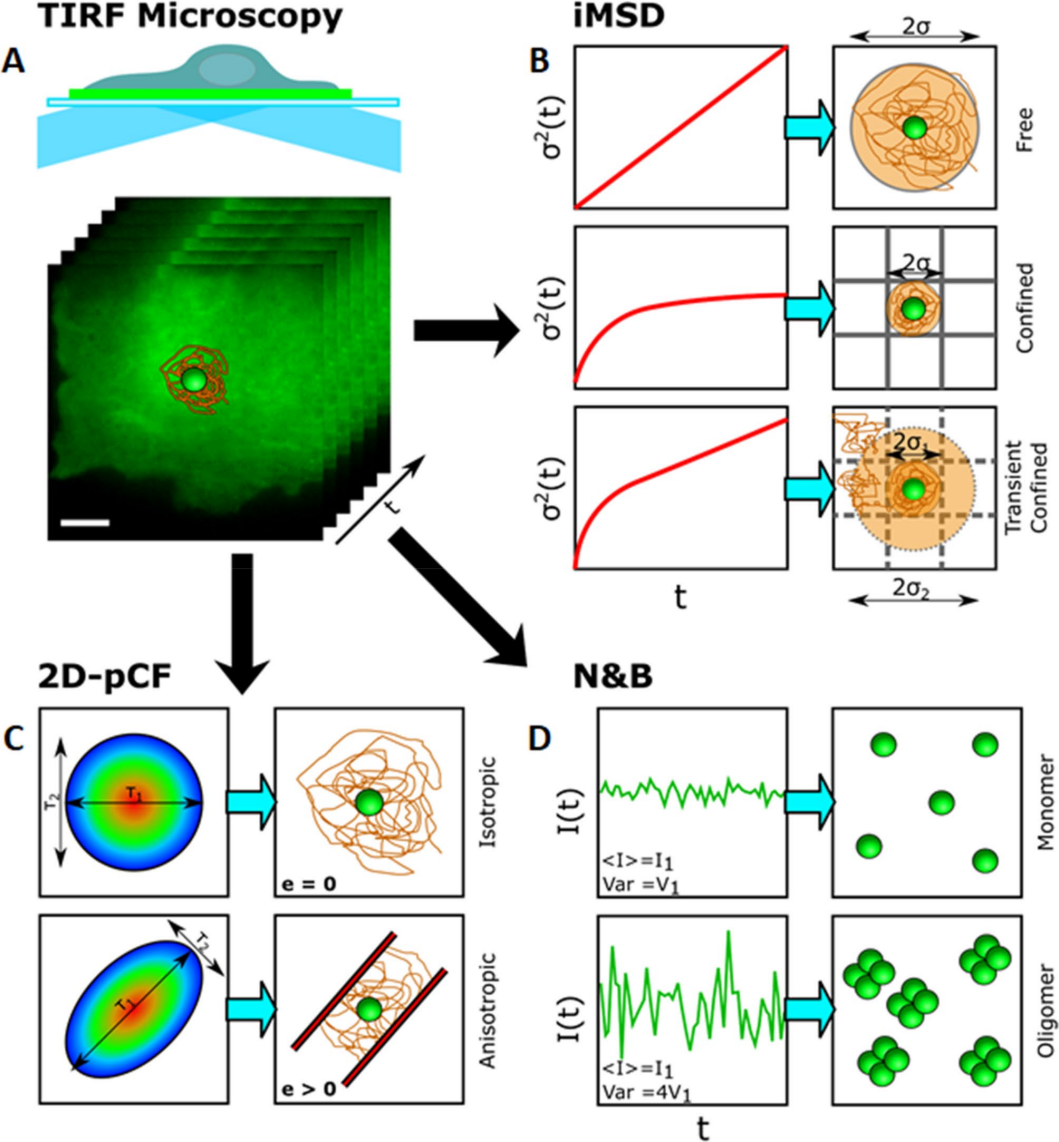
Schematic representation of the imaging and analysis approach. (**A**) Image sequences are acquired using a TIRF microscope. (**B**) Representative iMSD curves for different diffusion models (left) and their interpretation including free, confined and transient confined motions (right). (**C**) Example of local 2D pair correlation functions, 2D- pCF, (left) and their interpretation of either isotropic or anisotropic diffusion (right). (**D**) Temporal fluorescence fluctuation intensities for different oligomerization states (left) and their interpretation (right) obtained from Number and Brightness analysis.

Here, focusing on the KCNQ1-KCNE2 complex that is essential for normal gastric, thyroid and choroid plexus function, we employed a toolbox of complementary imaging and biophysical analysis techniques to probe heteromeric channel stoichiometry and subunit dynamics, designed to cover the entire spatial and temporal resolution required for a full picture of dynamic processes which are responsible for the formation of different complexes. Given the different expression levels and the diverse functional effects depending on the binding with specific subunits, it is not known how these complexes are regulated at the nanometer scale and what is the role of the nano-environment organization. A technique capable of measuring the modality and rate of lateral diffusion, stoichiometry and size of the confinement regions is needed to observe subtle changes in channels behavior by considering multiple parameters at the same time. Various methods used for studying these complexed proteins enabled observation of dynamic interactions between pairs of proteins, e.g. Förster resonance energy transfer (FRET) experiments. However, this method is restricted to protein pairs and can be influenced by factors such as dipole alignment and the local microenvironment. Advanced biophysical approaches, such as temporal fluctuation analysis, bypass the limitation of protein pairs and exploits the contribution of the variance of intensity fluctuations that reveals the distribution of higher oligomeric states in each pixel of an image. This method, the Number and Molecular Brightness (N&B) analysis, can be used to analyze image sequences acquired from laser scanning microscopes^36^ or camera based systems^37^. The advantage of these data is that the same image sequences can also be used to calculate the various types of protein mobility, diffusion rates of molecules and routes adopted by molecules at short- and long-range distances, using complementary fluorescence fluctuations-based approaches. For this work, fluorescence microscopy datasets were acquired with a total internal reflection (TIRF, Fig. 1A) microscope, which enables selective imaging of the plasma membrane of living cells, and the same dataset was analyzed with a combination of state-of-the-art fluorescence fluctuation spectroscopy (FFS) techniques. FFS techniques can be variously used to obtain important biophysical parameters such as protein size and concentration^38–43^, lateral diffusion coefficient^44–47^ and diffusion modality^46,48^, spatiotemporal heterogeneity in FRET^49,50^ and have also been applied in combination with super-resolution techniques^51–55^. In this work, we applied image-derived mean square displacement^56,57^ (iMSD, Fig. 1B), 2D pair correlation function^58^ (2D-pCF, Fig. 1C) and number and brightness analysis^36,37^ (N&B, Fig. 1D). These techniques allowed us to simultaneously obtain information about the organization of the nano-environment (iMSD), the protein directionality (2D-pCF) and oligomerization state (N&B) of the KCNQ1-KCNE2 complex. To better understand the dynamic properties of this potassium channel we performed our experiments by transfecting CHO cells with one of the subunits (mEGFP-tagged) or co-transfecting with both of them (mEGFP-, mCherry-tagged).

Our results reveal that the KCNE2 subunit shows either fast (> 0.02 µm^2^ s^−1^) or slow (< 0.02 µm^2^ s^−1^) micro-diffusion as well as increased diffusive eccentricity when co-expressed with KCNQ1 at short ranges (~ 220 nm). The nanodomain confinement size for the fast population matched that of KCNQ1, suggesting that this population may represent heteromeric KCNE2-KCNQ1 complex whereas the slow diffusive population represents the unbound KCNE2. The N&B analysis confirmed homodimers of KCNE2 proteins in the absence of KCNQ1 and tetramers upon binding to KCNQ1. Overall, we describe a model for KCNE2 with respect to KCNQ1 dynamic interactions at nanometer scale using advanced fluorescence fluctuation analysis.

## Results

### Fluorescent-tagged KCNQ1-KCNE2 channels exhibit normal channel activity.

**Figure 2:**
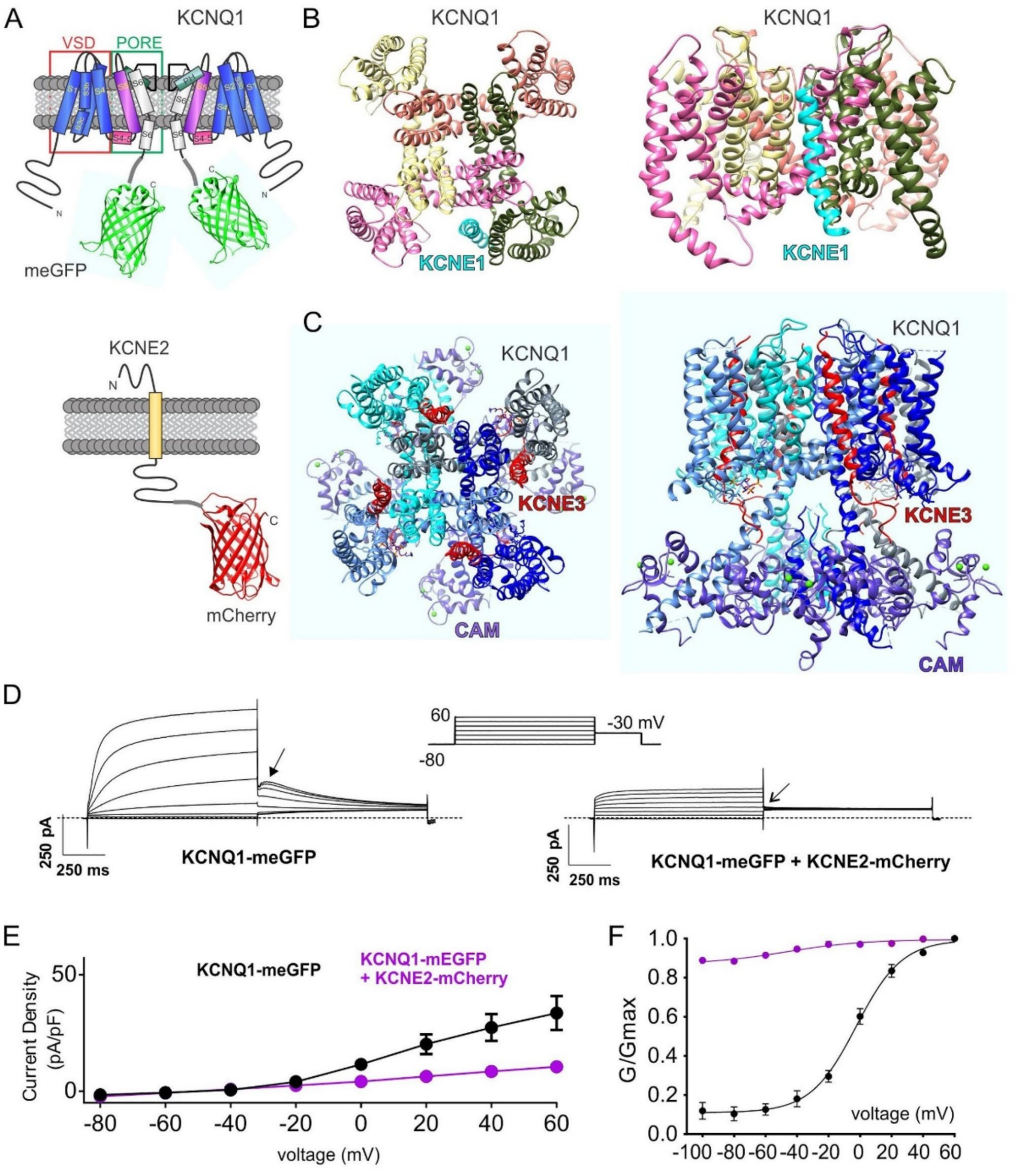
Fluorescent-tagged KCNQ1 and KCNQ1-KCNE2 express normal currents. (**A**) Transmembrane topologies of KCNQ1 and KCNE2 subunits showing fluorescent protein tagging of the cytosolic domains. (**B**) Extracellular (left) and intramembrane (right) views of structural model of KCNQ1-KCNE1 complex5. (**C)** Extracellular (left) and intramembrane (right) views of cryo-electron microscopy-resolved structure of KCNQ1-PIP2-KCNE3-calmodulin complex8. CAM = calmodulin. (**D**) Exemplary traces showing whole-cell patch-clamp recordings from CHO cells transfected with the subunit combinations shown. Dotted line indicates zero current level. Upper inset shows the voltage protocol. Arrows indicate tail current features. (**E**) Mean pre-pulse current density ± SEM for currents expressed by KCNQ1-meGFP alone (black) (n = 14) or with KCNE2-mCherry (purple) (n = 12). (**F**) Mean G/G_max_ relationship ± SEM measured from tail currents at arrows in panel D for currents expressed by KCNQ1-meGFP alone (black) (n = 14) or with KCNE2-mCherry (purple) (n = 12).

KCNQ1 α subunits each contain 6 transmembrane segments, S1-S6, organized into a VSD (S1-S4) and a pore module (S5-S6). We modified the human KCNQ1 cDNA such that the protein was C-terminally tagged with monomeric enhanced Green Fluorescent Protein (mEGFP). KCNE2 is a single transmembrane segment β subunit; we C-terminally tagged the human KCNE2 gene with genetically encoded mCherry (Fig. 2A).

There are no high-resolution structures or structural models for KCNQ1-KCNE2 channels. However, the Sanders group generated a high-resolution model of KCNQ1-KCNE1^32^, showing KCNE1 in a crevice between the VSD of one KCNQ1 α subunit and the pore module of a neighboring KCNQ1 α subunit (Fig. 2B). Previous stoichiometry studies argue for between two and four KCNE1 subunits in a KCNQ1-KCNE1 complex^33,34^. Using cryo-electron microscopy, the Mackinnon group solved the high-resolution structure of KCNQ1-KCNE3 channels with PIP_2_ and calmodulin bound^35^. The structure shows a tetramer of α subunits and four KCNE3 β subunits, positioned again between the VSD and pore modules of neighboring subunits (Fig. 2C). Here, we first expressed KCNQ1-mEGFP alone or with KCNE2-mCherry in Chinese Hamster Ovary (CHO cells) and recorded the currents generated using a standard voltage clamp protocol, with whole-cell patch clamp electrophysiology. KCNQ1-mEGFP expressed voltage-dependent currents with similar properties to those we and others previously observed for untagged KCNQ1, including a “hook” in the − 30 mV tail current that indicates recovery from inactivation^59^. Also similar to previous observations for untagged KCNQ1-KCNE2 channels, KCNQ1-mEGFP/KCNE2-mCherry channels were constitutively active, expressing smaller currents than homomeric KCNQ1-mEGFP (Fig. 2D,E) and with flat − 30 mV tail currents indicative of minimal voltage dependence and an absence of inactivation (Fig. 2F). Thus, the fluorescent tags used in this study did not noticeably perturb KCNQ1-KCNE2 electrical activity.

### iMSD provides dynamic fingerprinting of the nano-environment

Lateral modes of mobility at the plasma membrane can significantly impact protein signaling^60^. To this end, we applied iMSD analysis, which exploits spatiotemporal fluorescence fluctuations caused by protein diffusion to obtain various types of protein mobility reported by the iMSD^56,57^ curve. This curve can be used to define the diffusion model of a molecule and measure important biophysical parameters to obtain a dynamic picture of proteins diffusing in their nano-environment. iMSD is a particularly powerful technique since it yields similar information as single particle tracking (SPT)^61^ but is not limited by the need to visualize single molecules. This property allows to extend its applicability to more physiologically relevant applications and to a much larger range of protein concentrations.

**Figure 3:**
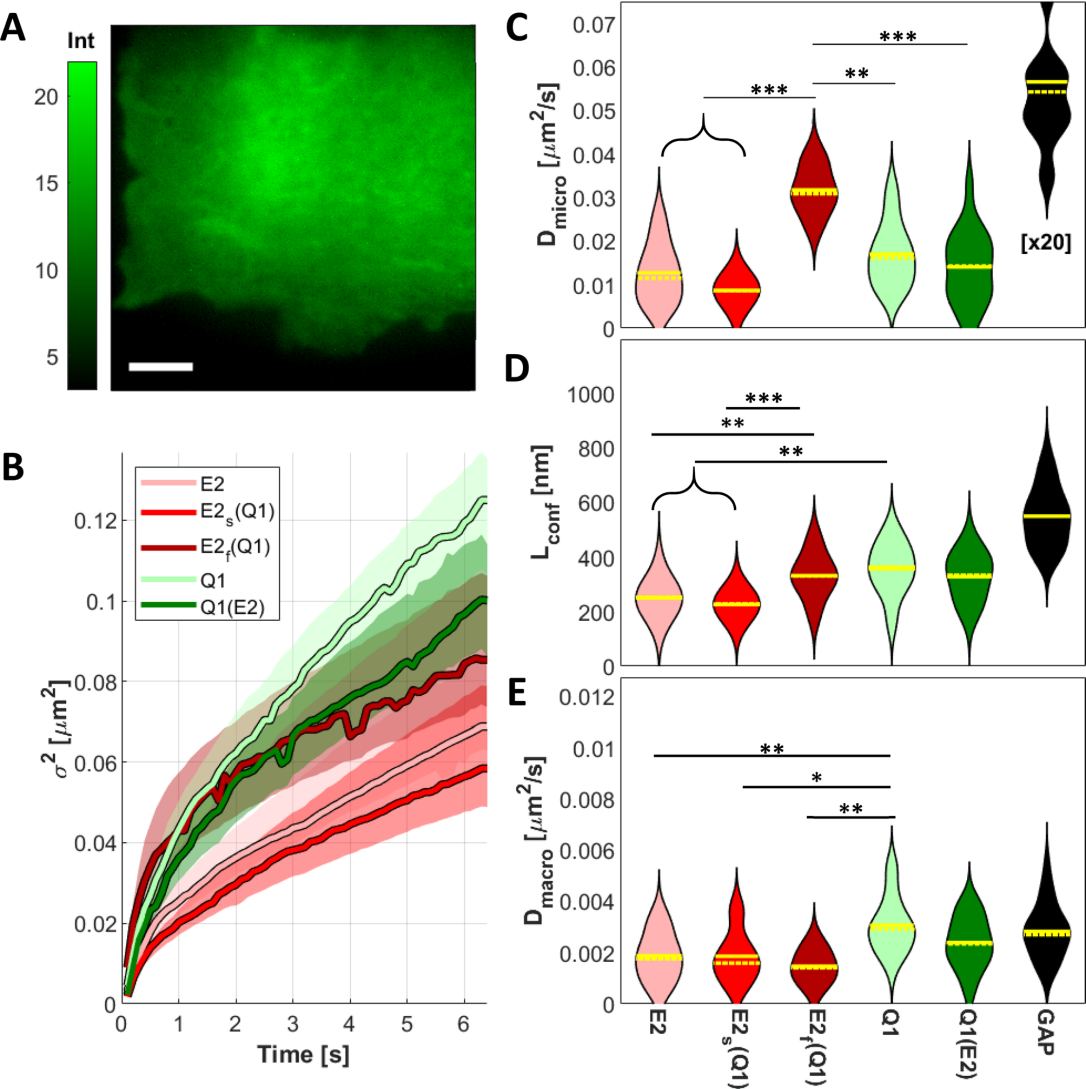
Representative average intensity image (**A**) of a CHO cell transfected with KCNE2-mEGFP. Average iMSD curves (colored curves) **(B)** with 95% confidence interval (shaded areas) for the conditions considered. Violin plots of the parameters obtained from the fitting with a transient confined model: D_micro_ (**C**), L_conf_ (**D**) and D_macro_ (**E**). The value of D_micro_ for GAP-mEGFP is 20 times the one shown in the graph (reported as [× 20]). Scale bar in A is 5 µm. Solid and dashed yellow lines represent the mean and the median of the distributions, respectively. Asterisks represent *P*-values < 0.05 (*), < 0.01 (**) and < 0.001 (***). *P*-values were obtained from Tukey's multiple comparison test. The conditions comprised in brackets are not statistically different (*P* > 0.5) among them but they are statistically significant compared to the other condition considered, with the displayed *P*-value.

In Fig. 3A, we show an example average intensity image of a CHO cell transfected with KCNE2-mEGFP construct. For each condition considered (summarized in Table 1) and for each cell of the dataset we computed its spatial autocorrelation function as a function of time $t$, we fitted it with a 2D Gaussian and plotted its variance as a function of time ${\sigma }^{2}\left(t\right)$, namely the iMSD function (Supplementary Fig. 1, right). The iMSD function was then fitted with three different diffusion models (free, confined or transiently confined diffusion) and calculated their residuals (an example in Supplementary Fig. 1, left) to determine the diffusion modality that best describes the dynamic behavior of the proteins expressed. From this analysis we determined that both KCNQ1 and KCNE2 have a transiently confined mode of diffusion in the membrane, whether expressed independently or co-expressed. The equation describing this diffusion modality is the following equation:

$$\sigma^{2} \left( t \right) = \frac{{L_{conf}^{2} }}{3}\left( {1 - e^{{ - \frac{t}{{\tau_{c} }}}} } \right) + 4D_{macro} t + \sigma_{0}^{2}$$

where ${\sigma }_{0}^{2}$ is an offset defined by the system’s optical resolution and ${\tau }_{c}$ is the confinement time. From the equation we were able to extrapolate the size of the domains in which the protein is diffusing (length of confinement,${L}_{conf}$) as well as the diffusion coefficient inside (micro-diffusion coefficient, ${D}_{micro}= \frac{{L}^{2}}{12{\tau }_{c}}+{D}_{macro}$) and across (macro-diffusion coefficient, D_macro_) the nano-domains for each cell.

**Table 1:** Summary of the experiment conditions and related terminology used throughout the paper.

Acronym	Proteins (co-)expressed
E2	KCNE2–mEGFP
E2(Q1)	KCNE2–mEGFP/KCNQ1–mCherry
Q1	KCNQ1–mEGFP
Q1(E2)	KCNQ1–mEGFP/KCNE2–mCherry
GAP	GAP-mEGFP

Interestingly, among the cells where KCNE2 was co-expressed with KCNQ1, we observed two distinct populations in the microdiffusion coefficient distributions (Supplementary Fig. 2): approximately 46% of the cells analyzed displayed an average D_micro_ slower than 0.02 µm^2^ s^−1^, while the rest of the cells displayed on average a faster diffusing population. Given this distinct behavior, we separated the cells displaying the slow and the fast micro-diffusion (named E2_s_(Q1) and E2_f_(Q1), respectively) in order to further investigate this finding.

In Fig. 3B we note that the behavior of E2_s_(Q1) largely overlaps with that of E2 alone, whereas E2_f_(Q1) has strong similarities with the diffusion behavior of Q1 and Q1(E2). For this reason, we hypothesize that E2_f_(Q1) represents the population bound to Q1, whereas E2_s_(Q1) represents the unbound population. As it can be appreciated in Table 2, the confinement length measured for E2_f_(Q1) and the Q1 conditions are comparable, suggesting that the two units reside in the same microenvironment, therefore supporting our hypothesis. Interestingly, the macro diffusion coefficient of E2_f_(Q1) is similar to E2_s_(Q1) and E2, suggesting that at longer spatiotemporal range E2_f_(Q1) is sensing the same environment of the unbound E2.

**Table 2:** List of iMSD parameters, values are mean $\pm$ standard deviation.

	E2	E2s(Q1)	E2_f_(Q1)	Q1	Q1(E2)
*Dmicro* (µm^2^ s^−1^)	0.0127 ± 0.0068	0.0087 ± 0.0019	0.0317 ± 0.0055	0.0170 ± 0.0074	0.0141 ± 0.0081
*Dmacro* (µm^2^ s^−1^)	0.0019 ± 0.0010	0.0019 ± 0.0010	0.0015 ± 0.0006	0.0031 ± 0.0011	0.0024 ± 0.0010
*Lconf* (nm)	250 ± 70	226 ± 43	332 ± 83	357 ± 94	329 ± 87

The microdiffusion coefficient distributions (Fig. 3C) revealed a similar average D_micro_ for KCNQ1 expressed alone or with KCNE2 co-expression, and a slower D_micro_ for KCNE2. The diffusion coefficient of E2_s_(Q1) is similar to that of the E2 samples, while E2_f_(Q1) shows a microdiffusion faster than Q1. From the analysis of the length of confinement of the diffusion domains (Fig. 3D), we observe that E2_f_(Q1) shows a L_conf_ similar to the Q1 and Q1(E2) samples, and bigger than E2_s_(Q1) nano-domains, which have a similar length as E2. As shown in Fig. 3E, E2f(Q1) and E2_s_(Q1) have a macro-diffusion similar to that of KCNE2 and statistically different from that of the Q1 samples. Complete tables of the *P*-values computed for the iMSD parameters are reported in Supplementary Fig. 3.

### Directionality of diffusion for KCNQ1 and KCNE2

Sensitivity to diffusion obstacles and their spatial organization can be an important factor affecting voltage-gated ion channels functionality. To understand if KCNQ1 or KCNE2 protein complexes experience diffusion barriers and if these interactions are unique to each complex, we used the 2D-pCF analysis^58^, which has the capability of mapping anisotropic paths at different spatial locations and therefore barriers to diffusion^62–65^. Briefly, each pixel in the image contains intensity fluctuations from the proteins occupied within that pixel when the image is captured. Sequential frame sequences are taken to obtain fluctuations as a function of time. To obtain the average time for a protein to move from one position (‘a’) to another position (‘b’) separated by a given number of pixels away, the fluctuation from position ‘a’ and position ‘b’ are cross-correlated by all possible delay times within in the image sequence. If the proteins diffuse into position ‘b’, a positive correlation will be detected with a characteristic decays time that took for the protein to reach that spot. To build a map of diffusion across the entire image, pixels are cross-correlated as function of time and in 24 angular directions. For each pixel of an image the pCF is calculated for all adjacent neighbor pixels in a radius around the pixel of interest at a given pCF distance δr. As an example, if a diffusing protein encounters an obstacle while transiting between the two pair-correlating points, its diffusion will no longer be isotropic but it will preferentially diffuse away from that obstacle and this will modify the time delay of the pair-correlation function, as depicted in Fig. 1C. The 2D-pCF analysis can be carried out at different distances in order to infer the presence of obstacle at short or long range.

To evaluate the directionality of diffusion of KCNQ1 and KCNE2, we extrapolated from the 2D-pCF analysis the values of eccentricity for five different pair-correlation distances. The eccentricity describes whether the protein is diffusing isotropically (e = 0) or anisotropically (e > 0).

**Figure 4:**
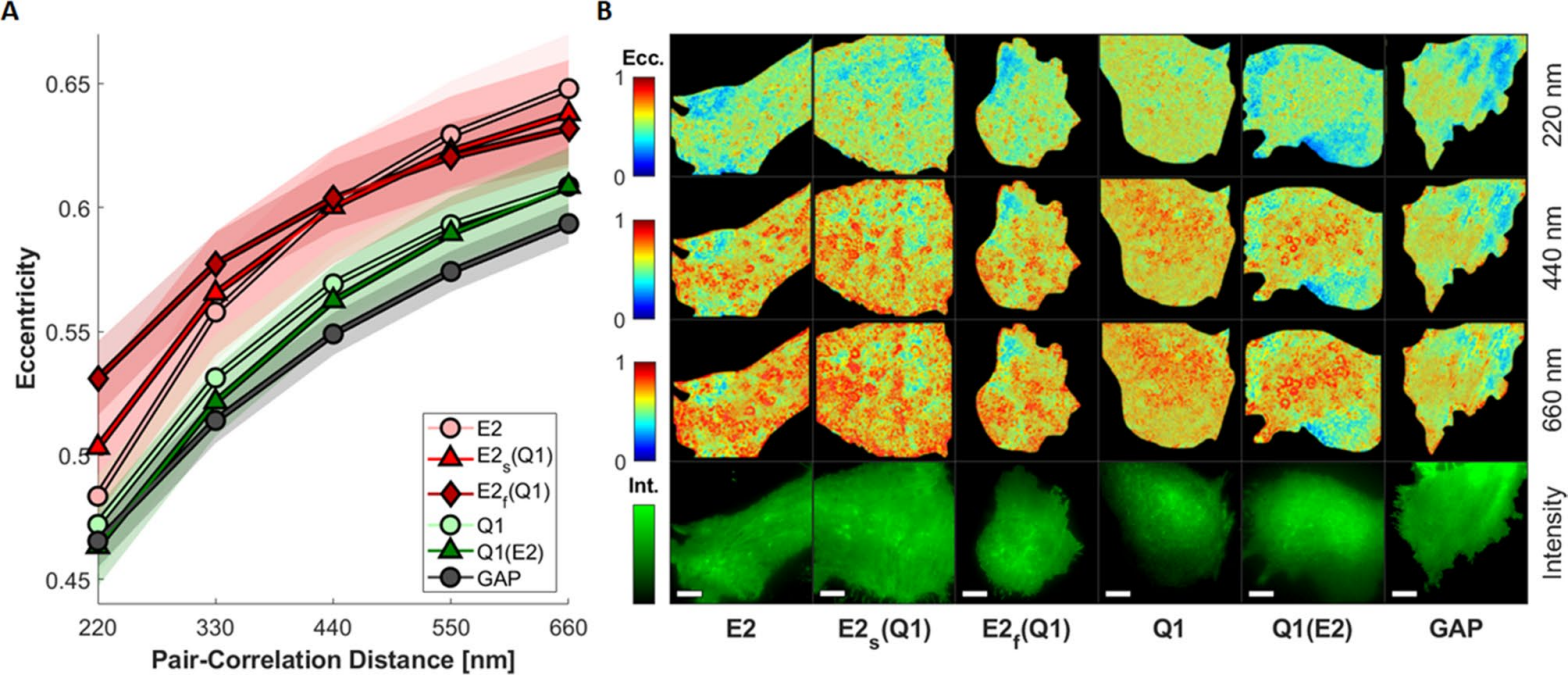
Median eccentricity as a function of the pCF distance considered (**A**), shaded areas represent the 95% confidence interval. **B**: Representative 2D-pCF eccentricity images computed at different distances and relative intensity images (**bottom**). Scale bar is 5 µm.

In Fig. 4A we show the average values of eccentricity as a function of the pair-correlation distance for all the conditions considered. As expected, for all the conditions the values of eccentricity become higher as the pair-correlation distance increase, since at larger distances there is a higher probability for the diffusing molecule to encounter obstacles. Our results show that the KCNQ1 channel, whether independently expressed or co-expressed, displays a low eccentricity, being comparable with that of our inert control membrane bound form of the mEGFP (GAP-mEGFP), which is not a functional protein and therefore its diffusion is solely dictated by the surrounding environment and is used here as a reference. KCNE2 generally shows a higher eccentricity, therefore a more anisotropic diffusion compared to Q1. At longer pair-correlation distances E2, E2_s_(Q1) and E2_f_(Q1) show a similar behavior, while at short range (220 nm) E2_f_(Q1) eccentricity significantly increases when compared to E2 and E2_s_Q1, suggesting a localization in membrane domains in which the directionality of the motion is more pronounced. Complete tables of the *P*-values computed for the 2D-pCF analysis for the average eccentricity and eccentricity at each distance are displayed in Supplementary Fig. 4.

Furthermore, from the 2D-pCF images (shown in Fig. 4B) it is possible to spatially map the eccentricity values for the different conditions and at the different pair-correlation distances. Compared to our inert control (GAP), we can see a strong heterogeneity of behavior in the cell membranes that increases with the distance. In particular we can observe the localization of binding loci at the membrane for both the KCNQ1 and the KCNE2, which appear as rings of high eccentricity^62^, as shown more in detail in Supplementary Fig. 6B.

### Number and Brightness analysis of KCNQ1-KCNE2 stoichiometry

**Figure 5:**
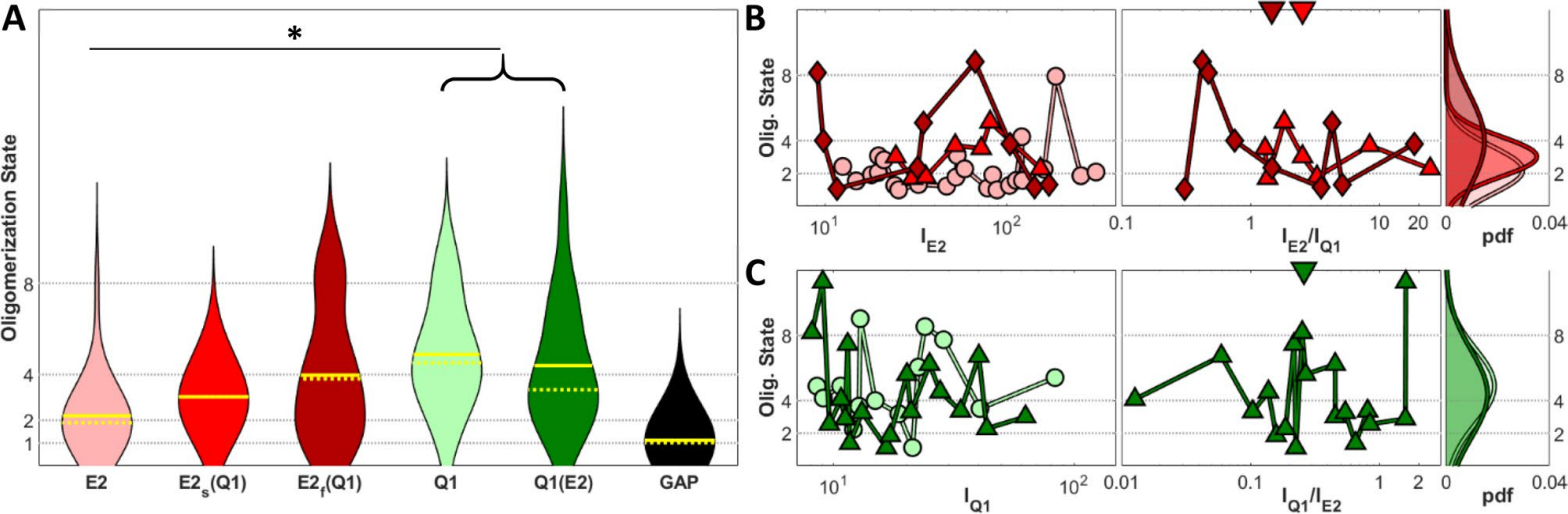
Violin plot for the oligomerization state as obtained by N&B analysis (**A**) for the samples considered. Dependence of the oligomerization state for the KCNE2-meGFP (**B**) and KCNQ1-meGFP (**C**) samples with the expression (left, represented by the average intensity of the meGFP channel) or co-expression (center, represented as the ratio between the average intensity of the meGFP and the mCherry channels) level, together with the corresponding probability density functions (right). Downward facing triangles in B and C represent the median co-expression level. Solid and dashed yellow lines represent the mean and the median of the distributions, respectively. Asterisks represent *P*-values < 0.05 (*), < 0.01 (**) and < 0.001 (***). *P*-values were obtained from Tukey's multiple comparison test. Symbols in **B** and **C** represent the median value obtained from a single cell. The conditions comprised in brackets are not statistically different among them (*P* > 0.5) but they are statistically significant compared to the other condition considered, with the displayed *P*-value. I_E2_ and I_Q1_ denote the average intensity of KCNE2 and KCNQ1, respectively.

Here, we applied N&B analysis to measure the oligomerization state of the KCNQ1-KCNE2 complex subunits and to further examine the differences between E2_s_(Q1) and E2_f_(Q1), using the membrane bound form of the mEGFP (GAP-mEGFP) as our monomeric reference. First, we found that the KCNE2 subunit exists on the membrane in the form of a dimer in the absence of KCNQ1 co-expression (Fig. 5A). Upon KCNQ1 co-expression, the E2_f_(Q1) results indicated four E2 subunits per KCNQ1-KCNE2 complex, whereas the E2_s_(Q1) suggested on average three E2 subunits (Fig. 5A). Since the trimeric form is not thought to be a physiological configuration, we interpreted our result as stemming from a mixture of dimeric (unbound) and tetrameric (KCNQ1-bound) forms of KCNE2. In this scenario, the slow micro-diffusion we measured for the E2_s_(Q1) is predominantly driven by the unbound dimeric population, although a small population of bound tetrameric KCNE2 is measured in the N&B analysis. Further indications to support the hypothesis that the E2_f_(Q1) represents the component bound to the KCNQ1 is the fact that the cells assigned to the E2_f_(Q1) show on average higher expression level of the KCNQ1 with respect to KCNE2 (Fig. 5B, center), compared to the E2_s_(Q1). N&B analysis of the KCNQ1 describes the complex as tetrameric with or without co-expression with KCNE2 (Fig. 5A,C). Importantly, from our data we could not correlate any dependence of the oligomerization state on the expression level (Fig. 5B-C), although it was reported elsewhere for KCNQ1-KCNE1 by some^33^ but not others^34^. This may be due to the fact that we are considering a limited range of expression levels, since we did not acquire very dim nor very bright cells in order to avoid low signal and saturation, respectively.

It’s worth noting that the width of the distribution is an intrinsic limitation of the N&B technique. In fact, the uncertainty in the determination of the oligomerization state scales with the number of oligomers, as reported elsewhere^36,66^. However, the samples considered were tested for normality and they were determined with high confidence (*P* > 0.91) to be Gaussian distributions, as expected. Moreover, both the width and the mean of the oligomerization state distribution for E2_f_(Q1) (Fig. 5B, right, dark red distribution) is comparable with the distributions shown for Q1 and Q1(E2) (Fig. 5B right, green distributions), which are known to be tetrameric. Complete tables of the *P*-values computed for the oligomerization state from N&B analysis are provided in Supplementary Fig. 5 and a representative map of brightness is shown in Supplementary Fig. 6C.

### Dynamic fingerprinting and nanoscale model of the KCNQ1-KCNE2 complex.

**Figure 6:**
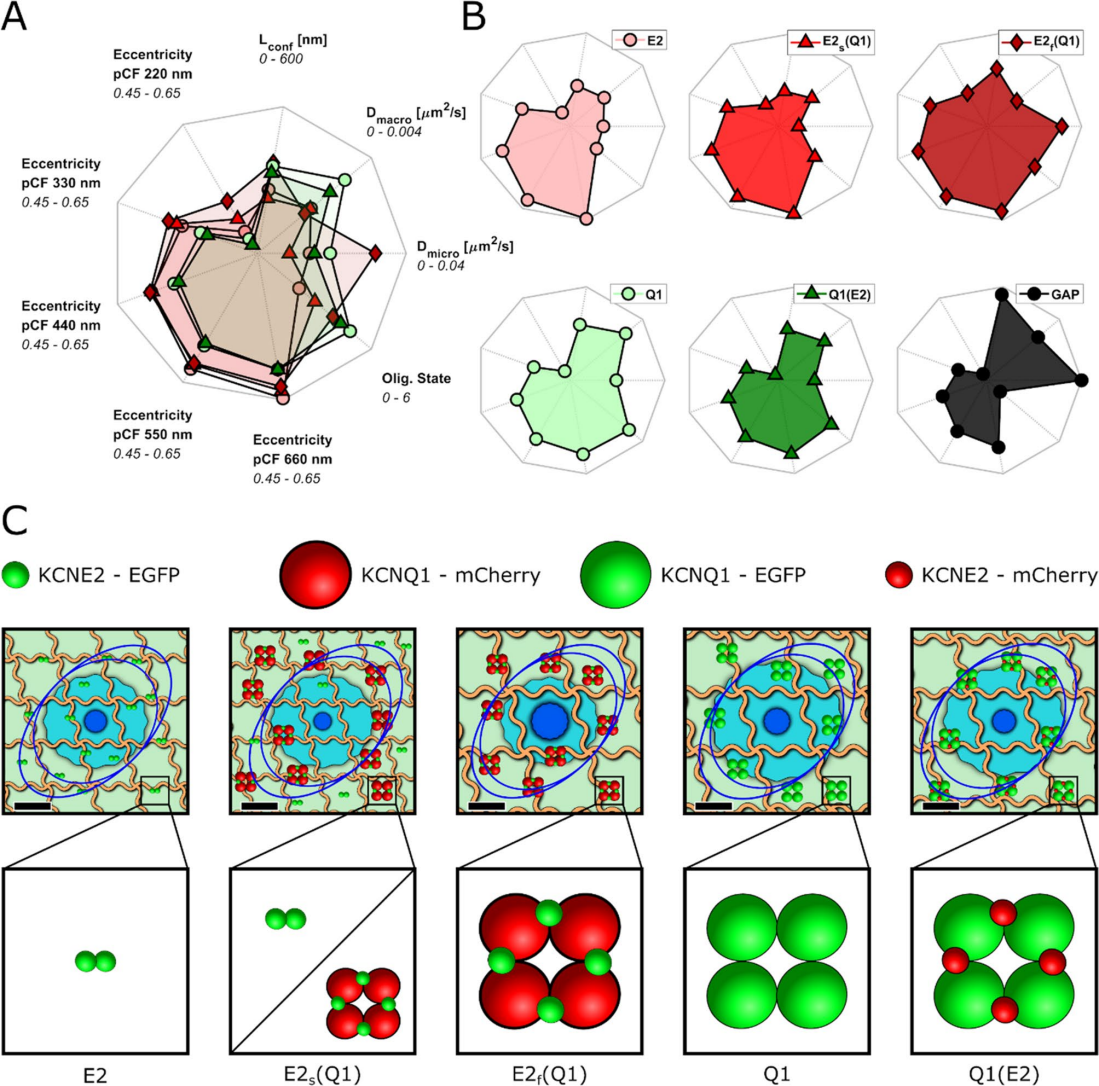
Combined (**A**) and individual (**B**) spider plots for all the conditions considered. (**C**) Comprehensive schematic representation of the diffusional nano-environment together with the model of the oligomerization state for the KCNQ1-KCNE2 complex. Scale bar for the model is 200 nm and proteins are shown 10 × their actual size. The dark blue and light blue circles represent the area covered by the diffusion of the protein in 0.5 s (calculated from *D*_*micro*_) and 60 s (calculated from *D*_*macro*_), respectively. Orange lines represent the nano-domains and the blue ellipses show the eccentricity as short scale (220 nm, internal ellipse) and long scale (660 nm, external ellipse).

To compare the biophysical and dynamic characteristics of KCNE2 and KCNQ1 alone or in complexes between them, we combined all the information of oligomerization and diffusion properties obtained by our multiplexed analysis and represented them in the form of a spider plot (Fig. 6A,B). This plot can provide an immediate quantification of the biophysical properties we can measure with our FFS-based approach. We used this plethora of information to create a graphic model of the membrane environment and homo- and hetero-oligomerization state of the proteins considered for this study (Fig. 6C). A complete table of the parameters used is available in Supplementary Table 1. In our model, the confinement regions are represented as square microdomains with size equal to L_conf_, the micro- and macro-diffusion as a circular region with diameter $d= \sqrt{4Dt}$ where $t$ is 0.5 s for D_micro_ and 60 s for D_macro_. Directionality is represented as two ellipses with eccentricity equal to the eccentricity calculated from 2D-pCF at a distance of 220 nm (inner ellipse) and 660 nm (outer ellipse). KCNQ1 and KCNE2 are represented with their oligomerization state measured from N&B and colored according to the appropriate fluorescent protein tagging. As shown pictorially by the dark blue circle in each panel, KCNE2 dimers diffuse slower and travel a shorter distance than when bound to tetramers of KCNQ1 (E2_f_(Q1)), approximately 150–200 nm and 300 nm in 0.5 s, respectively. When KCNE2 dimers are bound to tetramers of KCNQ1, two populations of distinct diffusions emerge as shown in Fig. 3. There are no notable differences in diffusion for complexes of KCNQ1 when bound or not bound to KCNE2.

## Discussion

In summary, we showed how the parallel implementation of multiple FFS techniques can give important insights into the diffusion behavior and oligomerization state of ion channels as well as the channel subunits stoichiometry. We provided a demonstration that the fluorescent tagging does not affect the electrophysiology measurements, from which we infer that the diffusional properties are conserved as well, and applied to the same dataset three analysis techniques to obtain information about micro- and macro-diffusion, confinement, directionality of motion, oligomerization state and stoichiometry of the KCNQ1-KCNE2 complex.

Analysis with iMSD lead to the discovery of a bimodal distribution in the D_micro_ parameter after co-transfection of the KCNE2 subunit with the KCNQ1 ion channel. We used this information to separate the diffusional behavior of the KCNE2 subunit in a slow and a fast component, which we hypothesized being the unbound and the Q1-bound population, respectively. Our hypothesis is supported by the fact that the slow component has the same length of confinement and oligomerization state as the KCNE2 transfected alone, whereas the fast component shows an increased oligomerization state as well as a confinement length comparable to KCNQ1, suggesting that they reside in a similar microenvironment. Counterintuitively, the bound component displays a faster microdiffusion, which is also associated with an increase in the confinement length, with respect to the unbound component. We attribute this behavior to a segregation of the unbound component in more confined and distinct domains in the cellular membrane, from which KCNE2 is released by binding to KCNQ1 or another intermediary. Interestingly, each cell appeared to contain either the slow or the fast population (but not both), which we showed were independent from (co-)expression levels (Supplementary Figs. 7–9), passage number or transfection. We speculate that other cell-to-cell variability in endogenous factors known to affect KCNQ1 and KCNE surface expression and interaction might explain this cell-specific behavior. Possible examples include Protein Kinase C activity, which regulates KCNQ1-KCNE surface expression and interaction^67–70^ or differential endogenous expression of proteins that interact with KCNE2, such as the focal adhesion protein Testin^71^. Other regulatory influences for diffusion of Kv channels include the underlying cytoskeletal structure, and interactions with other membrane proteins. Specifically, Kv2.1 and Kv1.5 have been detected to co-localize to caveolar and non-caveolar rafts enriched regions in cells^72^. Additional measurements have also shown that the function of Kv1.5 was altered upon cholesterol depletion and inhibition of sphingolipid synthesis. Moreover, lateral diffusion of voltage-gated sodium (Nav) channels is impacted by their ability to bind to the scaffolding protein ankyrin-G (AnkG), as long as the scaffold is in excess concentration versus the ion channel, and may regulate neuronal plasticity^65^. In addition to Nav channels, AnkG retains KCNQ2 and KCNQ3 subunits at the axon initial segment^73^. Further dedicated studies are needed to establish the precise underlying mechanisms controlling the diffusion of the KCNQ1-KCNE2 complex.

Our N&B analysis reported KCNE2 homodimers capable of reaching the plasma membrane without co-expression of KCNQ1. Our finding that KCNE2 reaches the plasma membrane when expressed alone, and forms homodimers in this environment, is in agreement with previous observations that non-fluorescent-tagged KCNE2 is able to traffic alone to the cell surface^74^. This observation is in contrast, however, with prior findings for non-tagged KCNE1, which was reported to be unable to reach the cell surface alone^75^. The KCNE2 regulatory subunit was previously found to traffic to the surface of HEK293 cells much more efficiently than KCNE1 or the hERG alpha subunit with which KCNE1 and KCNE2 can interact. Furthermore, KCNE2 was even found to be secreted into the extracellular medium. Importantly, KCNE2 proteins with the correct mass for dimers were detected in the extracellular fraction by western blot and confirmed by mass spectrometry analysis as being KCNE2 homodimers and the higher mass confirmed as not arising from glycosylation^74^. The dimeric form of KCNE2 reported by Um and McDonald^74^ was not tagged with a fluorescent molecule, supporting the premise that dimerization in our study is intrinsic to KCNE2 and not an artifact of tagging.

While the current study represents, to our knowledge, the first assessment of KCNQ1-KCNE2 channel stoichiometry, a number of studies have been conducted on related channels, primarily KCNQ1-KCNE1 (I_Ks_) complexes. Initial studies from the Goldstein lab, each employing different counting techniques (site-directed mutagenesis with macroscopic or microscopic functional analysis, and toxin binding), indicated a probable 4:2 KCNQ1-KCNE1 subunit stoichiometry, although the possibility of 4:4 was not entirely dismissed. The rigid 4:2 stoichiometry was also arrived at in a later study using a chemically releasable irreversible inhibitor. The Goldstein lab then subsequently utilized counting of fluorescent tag bleaching to further reinforce the idea of 4:2 KCNQ1-KCNE1 subunit stoichiometry and effectively rule out, in their hands, variable stoichiometry^34,76,77^. However, other labs have contended that they observe a variable stoichiometry with up to 4 KCNE1 subunits per channel complex, depending on KCNE1 expression levels relative to KCNQ1, based on single molecule fluorescence bleaching^33^. More recently, the MacKinnon lab solved a structure of KCNQ1-KCNE3 complexes using cryo-electron microscopy, and found a 4:4 stoichiometry^35^. In addition, using photobleaching, the Felipe lab recently counted 4:4 for KCNA3:KCNE4, although they too reported a variable KCNE4 stoichiometry in the complexes depending on the relative expression level, and concluded that functional attributes varied with stoichiometry^78^. Finally, using photobleaching, the D’Avanzo group concluded that complexes formed by pacemaker (HCN) channel alpha subunit and KCNE2, which may contribute to pacemaking in the heart and/or brain, have an expression-level dependent variable stoichiometry of between 4:1 and 4:4 (HCN:KCNE2)^79^.

The capability to assess the spatial distribution of multiple structural and functional parameters can have far-reaching applications in the study of the spatial organization of membrane channels. As an example, we report in Supplementary Fig. 6 an intensity image together with directionality and brightness maps of a cell displaying the slow KCNE2 population, in which it is possible to appreciate high brightness sites marked by a characteristic high eccentricity ring. Since CHO cells are not polarized and do not display any distinctive spatial feature (such as, e.g., the axon in neurons), the present study was conducted considering the overall median eccentricity and brightness of the whole cell, but may give important insights into the analysis of polarized cells such as gastric parietal cells, thyroid epithelial cells and choroid plexus epithelial cells, all cell types in which KCNQ1-KCNE2 channels play a crucial role.

## Conclusions

Our findings herein that KCNQ1-KCNE2 channels likely exist as octamers containing four of each subunit can guide our understanding of how these physiologically important channels function. Furthermore, given their role in several diverse, highly specialized cell types, the enhanced understanding of their movement within the cell membrane produced by this study can be built upon to understand the forces directing and localizing KCNQ1 and KCNE2 subunits in the various polarized cell types in which they are expressed, perturbation of which in vivo has been shown to contribute to complex disease states^23–25^.

The biophysical methods used in this work reveal the types of translational motion of molecules at the plasma membrane. Since the biophysical properties of both membrane organization and protein dynamics vary extensively across temporal and spatial scales, it is important to study the diffusion pattern which is dynamically kept out of equilibrium as cells tend to continuously regulate ion channel trafficking^80^. We showed that our multiplexed methods, and in particular iMSD and 2D-pCF analyses, can extract complex diffusion behaviors (Brownian diffusion, confinement/hopping dynamics) without the need to visualize single molecules, greatly simplifying sample preparation and analysis and providing unbiased and user-independent results while avoiding perturbating the correct functionality of the channels, as shown from our electrophysiology results. Furthermore, the N&B method provides information on the oligomerization state of the diffusing proteins, making the approach presented in this study ideal for a system of such complexity. In general, our use of TIRF imaging, a powerful yet relatively common technology, in combination with a series of Fluorescence Fluctuation Spectroscopy techniques allows researchers to determine, in a single acquisition, the oligomerization state, the diffusion modality and the nano-environment organization, making our approach generally suitable for a larger variety of applications in more than one field.

## Materials and methods

### Cell culture and transfection for whole cell patch-clamp

We seeded CHO cells (ATCC) onto poly-L-lysine treated glass coverslips and transfected using TransIT-LT1 (Mirus Bio LLC, Madison, WI, USA) the following day with CMV-based expression constructs containing cDNA for human KCNE2 (C-terminally mCherry-tagged), and/or KCNQ1 (C-terminally mEGFP-tagged). Cells were cultured in DMEM with 10% FBS and 1% penicillin/streptomycin in a 95% O_2_/5% CO_2_ humidified environment at 37 °C for 48–72 h post transfection prior to patch-clamping. We purchased cell culture consumables and reagents from VWR or Fisher Scientific unless otherwise stated.

### Whole-cell Patch Clamp

We recorded currents expressed in CHO cell as before^71^ using whole-cell patch-clamp at room temperature (22–25 °C) with 3–6 MΩ borosilicate glass electrodes backfilled with solution containing (in mM): 90 K Acetate, 20 KCl, 40 HEPES, 3 MgCl_2_, 1 CaCl_2_, 3 EGTA-KOH, 2 MgATP; pH7.2. We perfused cells continuously at 1–2 ml/min with an extracellular solution containing (in mM): 135 NaCl, 5 KCl, 5 HEPES, 1.2 MgCl_2_, 2.5 CaCl_2_, 10 glucose; pH 7.4. We purchased chemicals from Fisher Scientific or Sigma-Millipore. We held cells at − 80 mV in voltage clamp before applying the voltage step protocols and recording currents in response to pulses between − 80 mV and + 40 or + 60 mV at 20 mV intervals, followed by a single pulse to − 30 mV, using a CV − 7A Headstage (Axon Instruments, Foster City, CA, USA). Currents were amplified using a Multi-clamp 700B (Axon Instruments), low-pass filtered at 2–10 kHz using an eight-pole Bessel filter and digitization was achieved (sampling at 10–40 kHz) through a DigiData 1322A interface (Molecular Devices; Sunnyvale, CA). We used pClamp8 (Molecular Devices) Clampex software for data acquisition and Clampfit software for analysis, together with Graphpad Prism 7.0 (Graphpad; La Jolla, CA, USA).

### Cell culture and transfection for imaging

CHO (ATCC) cells were cultured in low glucose medium (Ham's F-12 K Medium Kaighn's modification, Gibco, ThermoFisher Scientific), supplemented with 10% v/v Fetal Bovine Serum (Heat Inactivated FBS, GenClone, Genesee) and 1% v/v Penicillin/Streptomycin Solution 100X (10,000 units of penicillin and 10 mg/mL streptomycin in 0.85% saline solution, GenClone, Genesee), in a 37 °C and 5% CO_2_ incubator. One day before transfection, cells were seeded in 35 mm glass bottom dishes (MatTek Corporation), previously coated with 2 µg/mL Fibronectin in Dulbecco's Phosphate Buffer Solution (Fibronectin human plasma 0.1% solution, Sigma-Aldrich; DPBS 1X without Ca, Mg, Phenol Red, GenClone, Genesee). CMV-based expression constructs containing cDNA for C-terminally mEGFP-tagged or mCherry-tagged human KCNE2 or KCNQ1 were used for single or co-transfections. For calibration, cells were transfected with GAP-mEGFP plasmid (pCAG-mEGFP was a gift from Connie Cepko, Addgene plasmid #14,757; http://n2t.net/addgene:14757; RRID: Addgene_14757). Transfections were performed with Lipofectamine 3000 Kit (Invitrogen, ThermoFisher Scientific) diluted in Opti-MEM I reduced-serum medium (Gibco, ThermoFisher Scientific), according to manufacturer instruction. Approximately one day after transfection, Opti-MEM medium was substituted with culture medium (low glucose medium supplemented with 10% v/v Fetal Bovine and 1% v/v Penicillin/Streptomycin Solution 100X). Cells were maintained in the incubator (37 °C and 5% CO_2_) prior to fluorescence microscopy experiments (48 h after transfection).

### Total Internal Reflection Fluorescence (TIRF) Microscopy

Fluorescence measurements were acquired with an Olympus IX83 TIRF. The system is equipped with a stagetop incubation system (Tokai Hit) providing CO_2_, humidity and temperature control. Temperature was maintained at 37 °C and CO_2_ at 5%. Samples were excited with a 491 nm laser (Olympus) for mEGFP or a SuperK Evo White Light Laser (NKT Photonics) with excitation filter 560/15 (Chroma) for mCherry and focused by a UAPO N 100x/1.49 NA Oil objective (Olympus). 512 × 512 images were collected with a pixel size of 55 nm. TIRF angle was set by software to achieve a penetration depth of 110 nm, corresponding to a TIRF angle of 67.91° for the 491 nm line and 69.45° for the 560 nm line. The fluorescence signal was separated by a 560 longpass dichroic beam splitter mounted on an Optosplit II (Cairn) and the two bands were collected by a Prime 95B sCMOS camera (Photometrics). Co-transfected samples were sequentially excited with the appropriate lasers. For the mEGFP channel, 1050 frames were acquired with 100 ms integration time, whereas for the mCherry channel 128 frames were acquired with 100 ms integration time. Laser power was set at 10% (green) and 60% (red) for all measurements. The microscope was controlled by Micromanager 1.4 (ImageJ). A sample of Tetraspeck fluorescent beads (100 nm diameter) were used to calibrate the point spread function waist and for fluorescence channels registration.

### Analysis

The number of cells analyzed for each condition and for each analysis as described below are listed in the following table:

**Table:** 

*Experiment*	*Number of cells*
E2	23
E2_s_(Q1)	12
E2_f_(Q1)	14
Q1	22
Q1(E2)	23
GAP	74

#### 2D-pCF

2D-pCF analysis was performed with SimFCS 4 (Globals). For each cell a user-defined mask was obtained and used in the analysis. Sampling parameters such as pixel size (55 nm), frame time (100 ms) and point spread function waste (210 nm) were entered in the program. 2D-pCF analysis was performed at 4, 6, 8, 10 and 12 pixels of distance.

#### iMSD

iMSD analysis was performed with a custom code written in MATLAB. From a user-defined rectangular region the spatiotemporal ACF, represented by a 3D matrix, was computed. Each XY plane of this matrix was fitted to a two-dimensional Gaussian function and the σ^2^(t) was stored and fitted with a free diffusion, confined and transient confined models, as described by the following equations:

$${\text{Free}}\;{\text{diffusion}}:\sigma^{2} \left( t \right) = 4D_{micro} t + \sigma_{0}^{2}$$

$${\text{Confinement}}:\sigma^{2} \left( t \right) = \frac{{L_{conf}^{2} }}{3}\left( {1 - e^{{ - \frac{t}{{\tau_{c} }}}} } \right) + \sigma_{0}^{2}$$

$${\text{Transient}}\;{\text{confinement}}:\sigma^{2} \left( t \right) = \frac{{L_{conf}^{2} }}{3}\left( {1 - e^{{ - \frac{t}{{\tau_{c} }}}} } \right) + 4D_{macro} t + \sigma_{0}^{2}$$

#### N&B

Number and brightness analysis was performed with a custom code written in MATLAB. The mean and variance along the temporal dimension of the image stack was computed and the brightness was obtained after correcting for the dark noise/offset and the gain of the camera, as described elsewhere^37,53,66^. The oligomerization state was corrected to account for protein maturation and misfolding as described elsewhere^81^. The brightness of the monomers was found to increase linearly with the average intensity; therefore, we performed a fitting of the brightness of all the monomers acquired as a function of the average intensity. The resulting function was used to obtain the oligomerization state for the experiments, scaled with the appropriate average intensity value. Bleaching was not observed and therefore no correction was performed.

### Statistical analysis

#### Electrophysiology

All values are expressed as mean ± SEM. Students’ t-test was used for statistical comparisons. All P-values were two-sided. Statistical significance was defined as P < 0.05.

#### FFS

Distributions are represented with violin plots using the MATLAB function “Hoffmann H, 2015: violin.m—Simple violin plot using MATLAB default kernel density estimation. INRES (University of Bonn), Katzenburgweg 5, 53,115 Germany.“ Bandwidth was set as 0.0033 (Fig. 3C), 53.33 (Fig. 3D), 0.0005 (Fig. 3E) and 1.5 (Fig. 5A). Tukey’s test was used for multiple comparisons statistics by using the MATLAB function “multcompare” with statistics provided by the “anova1” function, which computes the one-way ANOVA (analysis of variance). Statistical significance is shown as asterisks corresponding to *P*-values < 0.05 (*), < 0.01 (**) and < 0.001 (***). All distributions have been tested for normality by a single sample Kolmogorov–Smirnov test, yielding high confidence results for all distributions (*P *> 0.91). Complete diagrams of statistical significance are shown in the Supplementary Figures.

## Supplementary Information

Supplementary Information.

## Data Availability

The datasets generated during and/or analyzed during the current study are available from the corresponding authors on reasonable request.
