# Supplementary material for: Fluorescence Fluctuation Spectroscopy enables quantification of potassium channel subunit dynamics and stoichiometry
Source: Sci Rep. 2021 May 21;11:10719. doi: 10.1038/s41598-021-90002-2 (PMC8140153; doi:10.1038/s41598-021-90002-2)
Supplement: Supplementary file 1 — Supplementary Information. [file 41598_2021_90002_MOESM1_ESM.docx]

**Fluorescence Fluctuation Spectroscopy enables quantification of potassium channel subunit dynamics and stoichiometry**

**Giulia Tedeschi^1†^, Lorenzo Scipioni^1†^, Maria Papanikolaou^2^, Geoffrey W. Abbott^2^*, Michelle A. Digman^1^***

^1^ Department of Biomedical Engineering, Laboratory for Fluorescence Dynamics, University of California Irvine, Irvine, CA 92697, USA.

^2^ Department of Physiology and Biophysics, Bioelectricity Laboratory, School of Medicine, University of California Irvine, Irvine, CA 92697,USA.

Corresponding authors:
*Dr. Geoffrey W. Abbott
Department of Physiology and Biophysics, Bioelectricity Laboratory, School of Medicine, University of California Irvine, Irvine, CA 92697,USA.
**Email:** [abbottg@uci.edu](mailto:abbottg@uci.edu) **Phone:** 949 824 3269

*Dr. Michelle A. Digman
Department of Biomedical Engineering, Laboratory for Fluorescence Dynamics, University of California Irvine, Irvine, CA 92697, USA.
**Email:** [mdigman@uci.edu](mailto:mdigman@uci.edu) **Phone:** 949 824 3255

^†^These authors contributed equally to this work

**SUPPLEMENTARY FIGURES**

**
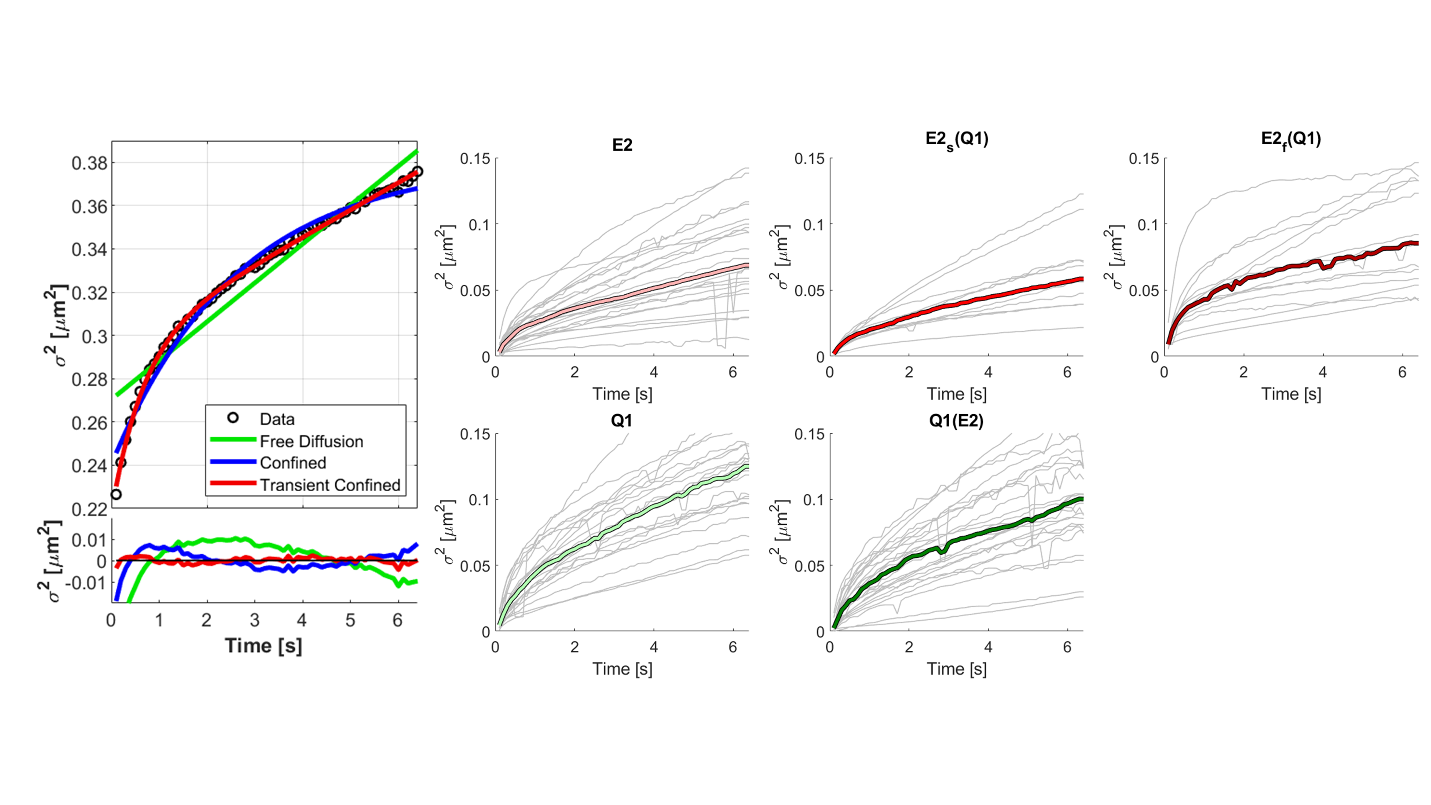
**

***Supplementary Figure 1***: *Example of* *iMSD curve fitted with three different diffusion models and their residuals (left), corresponding to the cell depicted in* ***Figure 3.*** *iMSD* *Curves corresponding to the single experiments (gray lines) and the corresponding mean curve (colored) for the five conditions considered (right).*

**
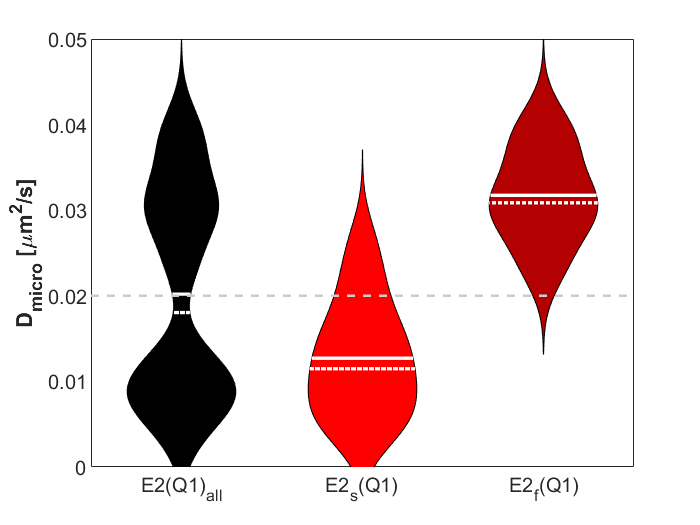
**

***Supplementary Figure 2***: *Rationale for defining two population for the co-expressed E2-mEGFP and Q1-mCherry condition. The D_micro_ distribution of all cells (black) shows a clearly bimodal distribution, the mean of which is 0.02 µm^2^s^-1^, value that was used to separate the two populations: E2_s_(Q1), depicted in red and E2_f_(Q1), depicted in dark red.*


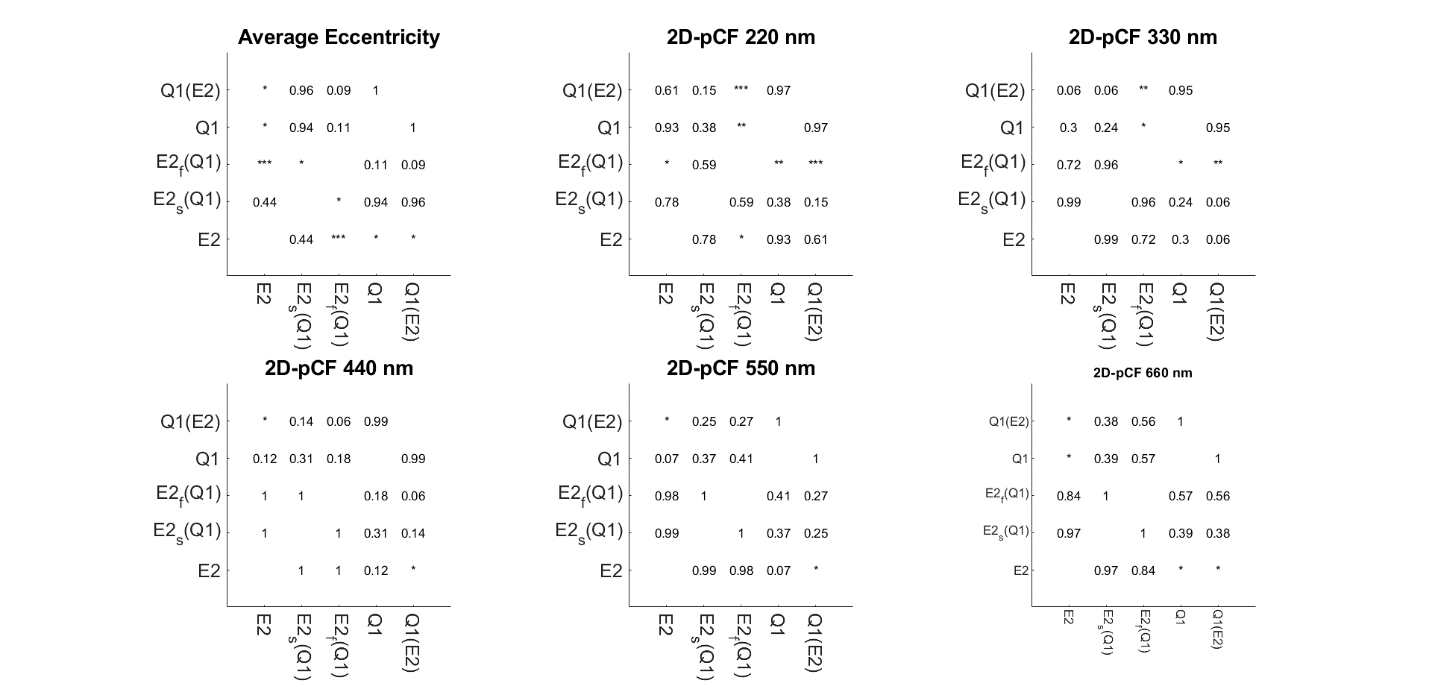

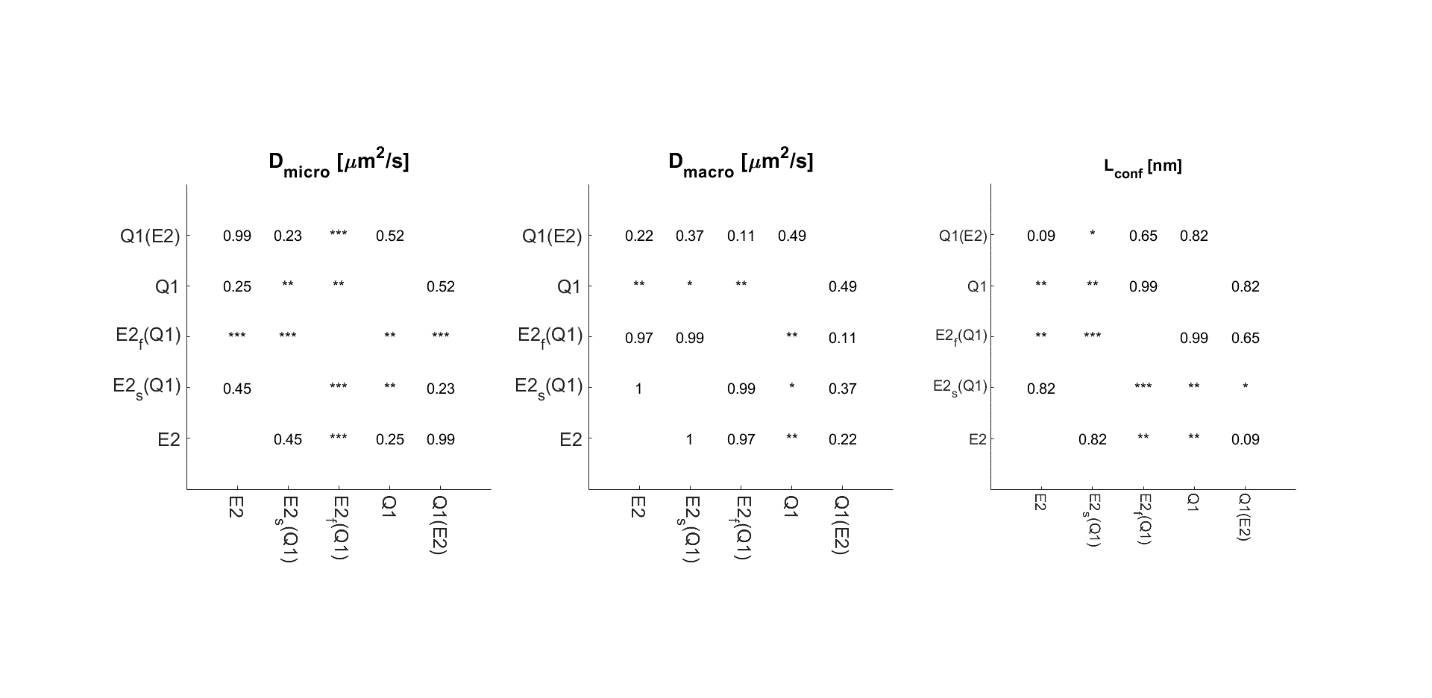


***Supplementary*** ***Figure 4***: *Table of the p-values computed for the 2D-pCF analysis for all cases: Average eccentricity (top left) and eccentricity at each distance are displayed. Asterisks represent p-values <0.05 (*), <0.01 (**) and <0.001 (***)*

***Supplementary Figure 3:*** *Table of the p-values computed for the iMSD parameters for all cases: D_micro_ (left), D_macro_ (center) and L_conf_ (right). Asterisks represent p-values <0.05 (*), <0.01 (**) and <0.001 (***).*


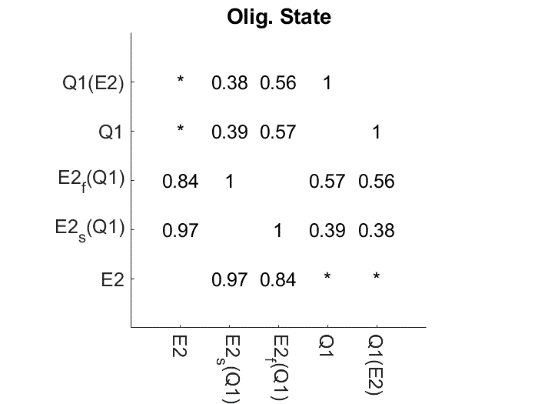


***Supplementary Figure 5:*** *Table of the p-values computed for the oligomerization state from N&B analysis for all cases. Asterisks represent p-values <0.05 (*), <0.01 (**) and <0.001 (***)*

***Supplementary Figure 6****:* *Intensity (A), 2D-pCF eccentricity at 220 nm (B) and Brightness (C) images of the same cell, corresponding to a E2_s_(Q1) sample. Top images represent the entire dataset, whereas the bottom images represent a region of interest (ROI) described by the black squares.*

*
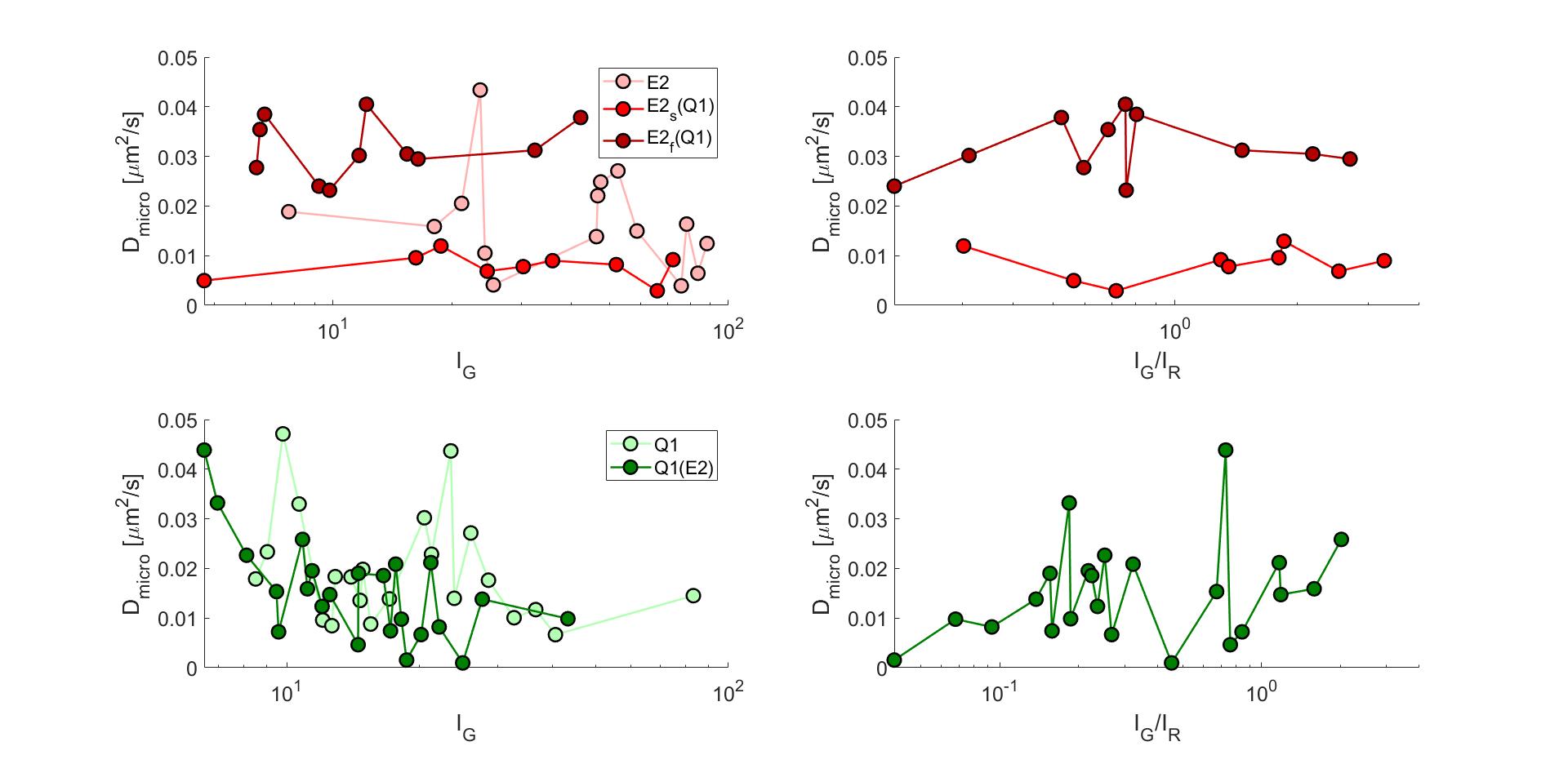
*
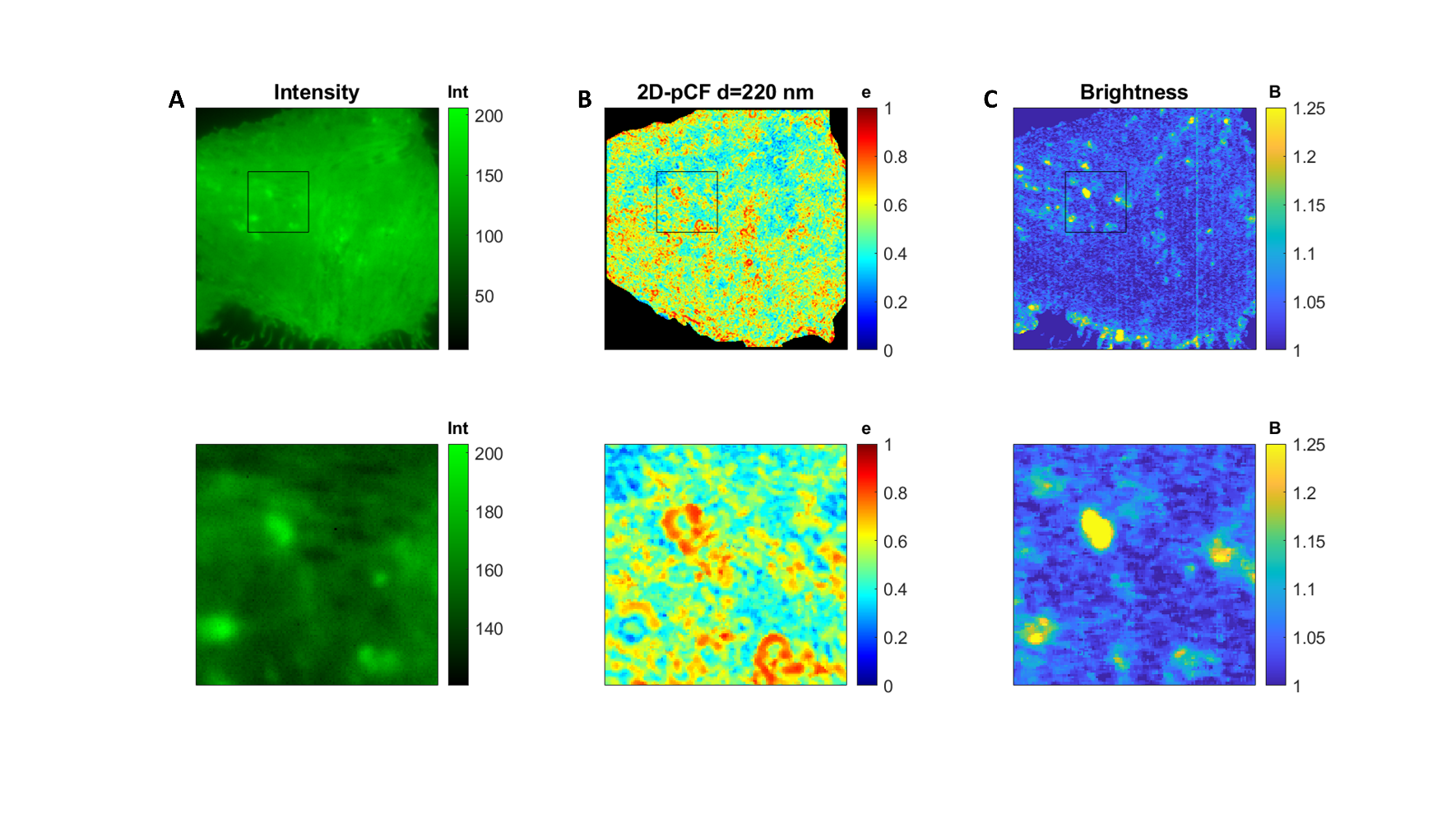


***Supplementary Figure 7:*** *Dependence of the D_micro_ from the expression (left) and co-expression (right) levels for the E2 (top) and Q1 (bottom) samples. I_G_ and I_R_ denote the average intensity in the green (mEGFP) and the red (mCherry) channel, respectively.*


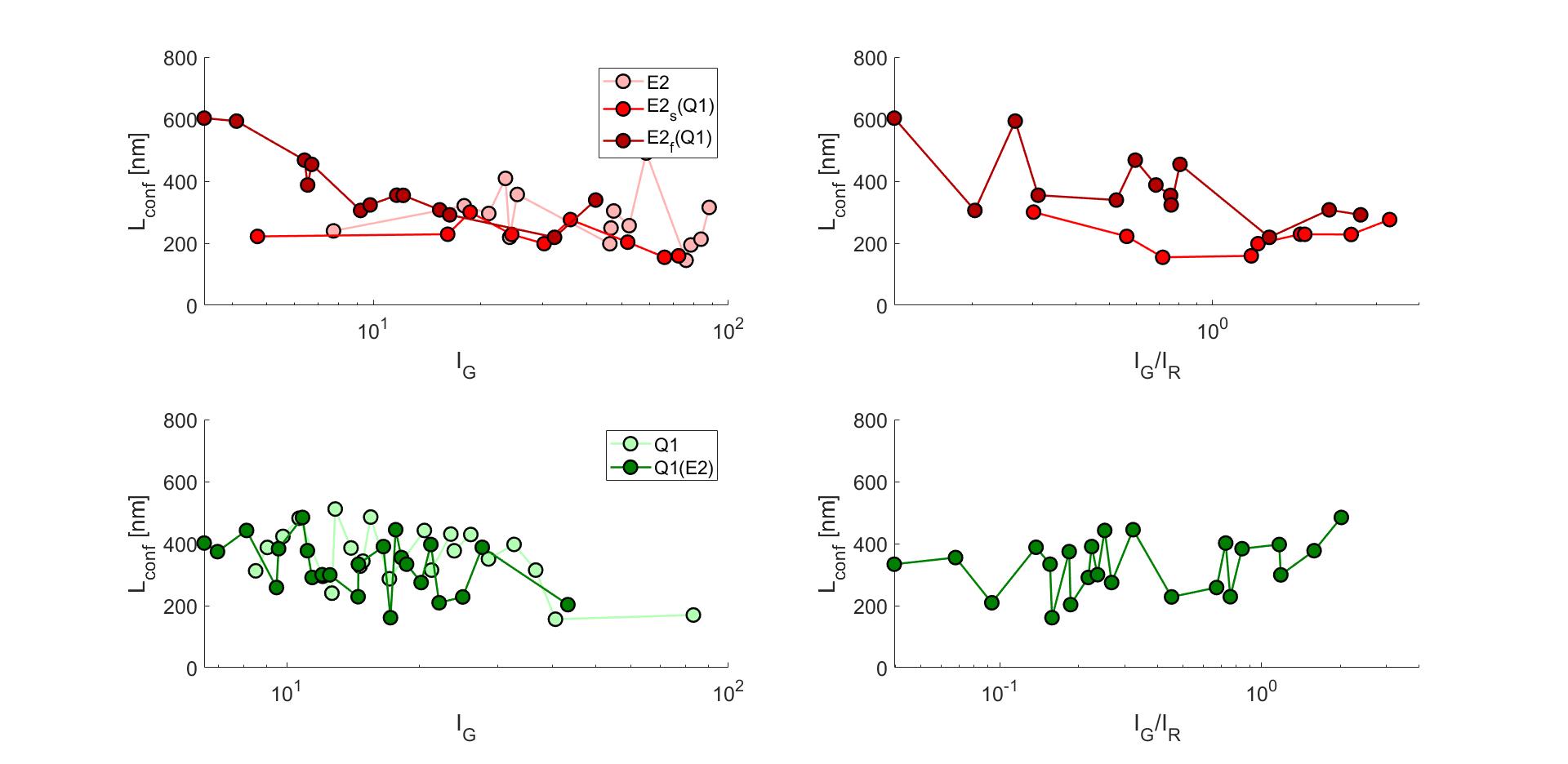


***Supplementary Figure 8:*** *Dependence of the L_conf_ from the expression (left) and co-expression (right) levels for the E2 (top) and Q1 (bottom) samples. I_G_ and I_R_ denote the average intensity in the green (mEGFP) and the red (mCherry) channel, respectively.*

*
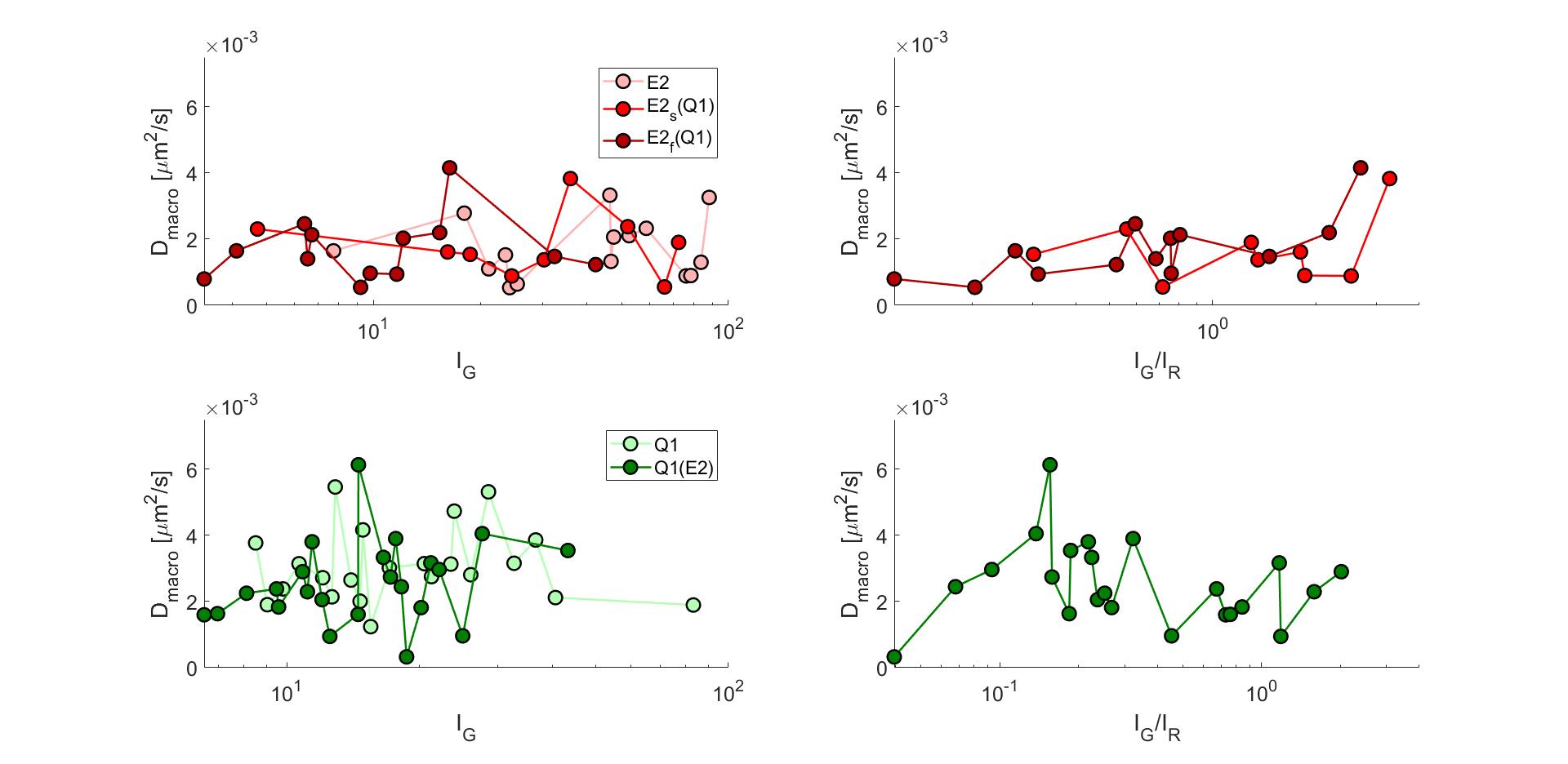
*

***Supplementary Figure 9:*** *Dependence of the D_macro_ from the expression (left) and co-expression (right) levels for the E2 (top) and Q1 (bottom) samples. I_G_ and I_R_ denote the average intensity in the green (mEGFP) and the red (mCherry) channel, respectively.*

|  | **d_micro_ (0.5s) [nm]** | **d_macro_ (60s) [nm]** | **e_220_** | **e_660_** | **L_conf_ [nm]** | **Oligomerization State** |
| --- | --- | --- | --- | --- | --- | --- |
| **E2** | 189 | 758 | 0.47 | 0.65 | 261 | Dimers |
| **E2_s_Q1** | 148 | 754 | 0.5 | 0.64 | 226 | Dimers/Tetramers |
| **E2_f_Q1** | 318 | 712 | 0.54 | 0.63 | 371 | Tetramers |
| **Q1** | 223 | 969 | 0.46 | 0.61 | 357 | Tetramers |
| **Q1E2** | 198 | 889 | 0.46 | 0.61 | 329 | Tetramers |

***Supplementary Table 1:*** *Biophysical parameters used to construct diffusion model described in* ***Figure 6****.*
